# *Pinus koraiensis* Essential Oil Attenuates the Pathogenicity of Superbacteria by Suppressing Virulence Gene Expression

**DOI:** 10.3390/molecules29010037

**Published:** 2023-12-20

**Authors:** Ji-Hee Kim, Young-Hoi Kim, Bog-Im Park, Na-Young Choi, Kang-Ju Kim

**Affiliations:** 1 Department of Convergence Technology for Food Industry, Wonkwang University, Iksan 54538, Republic of Korea; ckhae@naver.com; 2 Transdisciplinary Major in Learning Health Systems, Department of Health and Safety Convergence Science, Graduate School, Korea University, Seoul 02841, Republic of Korea; 3 Department of Food Science and Technology, College of Agriculture and Life Sciences, Jeonbuk National University, Jeonju 54896, Republic of Korea; yhoi1307@hanmail.net; 4 Department of Food and Nutrition, Kunsan National University, Kunsan 54150, Republic of Korea; parkbogim@hanmail.net; 5 College of Education, Wonkwang University, Iksan 54538, Republic of Korea; 6 Department of Oral Microbiology, School of Dentistry, Wonkwang University, Iksan 54538, Republic of Korea

**Keywords:** *Pinus koraiensis*, superbacteria, MRSA, virulence gene, antibacterial

## Abstract

In the quest to combat infections attributable to antibiotic-resistant superbacteria, an essential oil derived from the needles of *Pinus koraiensis* Sieb. et Zucc. (PKEO) has emerged as a promising solution. In this study, we demonstrate that PKEO can be used to inhibit the growth, glucose metabolite acidogenicity, and biofilm formation of methicillin-resistant *Staphylococcus aureus* (MRSA). Quantitative PCR analysis provided direct evidence that PKEO reduces the mRNA expression of the accessory gene regulator A (*agrA*) and staphylococcal accessory regulator A (*sarA*), thereby indicating its inhibitory effect on pathogenic regulatory genes. Chromatographic analyses of PKEO identified terpene hydrocarbons as prominent essential oil constituents. These compounds, notably α-pinene, limonene, and β-caryophyllene, have been established to have antimicrobial properties. Our findings indicate that an oil derived from *P. koraiensis* can effectively combat antibiotic-resistant strains by disrupting the pathogenicity regulatory system, thereby establishing PKEO as a promising candidate for the treatment of MRSA infections.

## 1. Introduction

In recent decades, the excessive use or misuse of antibiotics has led to the emergence of so-called superbacteria that are resistant to conventional antibiotics and pose a considerable threat to the health of infected individuals. A well-established group of superbacteria are strains of methicillin-resistant *Staphylococcus aureus* (MRSA), which are non-responsive to methicillin and other beta-lactam antibiotics. MRSA bacteremia, which can be accompanied by complications such as infective endocarditis, bone infection, and septicemia [1,2], is becoming increasingly prevalent and associated with heightened fatality [3]. According to the 2019 Antibiotic Resistance Threats Report in the United States, more than 80,000 cases of severe MRSA infections have occurred annually since 2013, resulting in over 11,000 fatalities [4]. Furthermore, the European Center for Disease Prevention and Control found that 13 of 44 European countries had MRSA infection rates in excess of 25% [5], and the 2021 China Antimicrobial Resistance Surveillance Results Report indicated a nationwide average detection of 29.4% for MRSA infections [6].

Multiple antibiotic treatments have been selectively administered for the treatment of MRSA infections. Among these, vancomycin has been specified by the Infectious Diseases Society of America as a typical medication for treating ailments such as MRSA-related bacteremia, infective endocarditis, pneumonia, and meningitis [7]. However, when administered for prolonged periods exceeding 2 weeks, conventional vancomycin therapy can lead to a 30% incidence of acute renal failure due to nephrotoxicity, and it can also trigger a hypersensitivity reaction referred to as Red Man syndrome [8,9,10]. Since the report of vancomycin-resistant *Staphylococcus aureus* (VRSA) cases in the United States in 2002, there has been an increasing trend in the prevalence of this infection [11,12,13]. Among other antibacterials, linezolid, an FDA-approved medication for the treatment of MRSA-related skin and soft tissue infections, as well as pneumonia, is associated with the occurrence of thrombocytopenia [7,14], whereas when administered at high concentrations, daptomycin, which is used to treat MRSA bacteremia, infective endocarditis, and skin infections, can result in reduced renal clearance, leading to increased plasma concentrations and potential side effects, such as elevated creatine kinase levels [7,15]. Given the limitations and increasing loss of efficacy of conventional antimicrobials, essential oils, derived from medicinal plants rich in secondary metabolites with proven safety and pharmacological efficacy, have garnered increasing attention as prospective alternatives for combating severe MRSA infections and as a means of mitigating the side effects associated with conventional antibiotics [16,17].

*Pinus koraiensis* Siebold and Zuccarini (*P. koraiensis*) is a widely distributed perennial evergreen tree in the family Pinaceae found growing in Korea, northeastern China, the Russian Far East, and central Japan [18,19]. In classical Chinese medicine, *P. koraiensis* has a long-standing history of use as a medicinal herb, and, in Korea, is used as an herbal remedy with antiseptic, diuretic, and analgesic properties, which is typically applied in the treatment of burns and skin ailments [18,20]. Previous studies have established that an essential oil of *P. koraiensis* (PKEO) has anticancer, antioxidant, antifungal, and antibacterial properties [21,22,23,24], thereby indicating that this oil could have potential pharmacological efficacy against severe MRSA infections. However, despite its established antibacterial properties, there has been a lack of research focusing on the association between PKEO and the virulence factors of MRSA. In this study, we identified the key chemical constituents of PKEO using gas chromatography–flame ionization detection (GC–FID) and gas chromatography–mass spectrometry (GC–MS) and examined the effects of PKEO on MRSA growth, acidogenicity, and the expression of the pathogenicity determinants *agrA* and *sarA*.

## 2. Results

### 2.1. Chemical Composition of PKEO

In this study, we used a hydro-distillation method to prepare an essential oil from the needles of *P. koraiensis*, obtaining a yield of 0.52% on a weight basis. Using chromatographic techniques, we analyzed the phytochemical profile of the oil and determined the contents of terpene hydrocarbons. The essential oil of *P. koraiensis* (PKEO), which was characterized by a pale yellow color and pine odor, was established to comprise 51 constituents, accounting for a total of 96.47% of its composition (Table 1). GC–FID and GC–MS analyses revealed that the PKEO mainly comprised terpene hydrocarbons, with a predominance of monoterpenoid compounds (72.24%), including α-pinene (21.32%), α-terpineol (11.03%), δ-3-carene (10.32%), terpinolene (7.20%), camphene (6.22%), limonene (5.17%), myrcene (4.60%), bornyl acetate (3.85%), and β-pinene (2.53%). Of the remaining compounds, the sesquiterpenoids β-caryophyllene (4.69%) and δ-cadinene (4.38%) accounted for 9.07% of the total GC peak area.

### 2.2. PKEO Suppresses MRSA Growth and Acidogenicity

**Figure 1:**
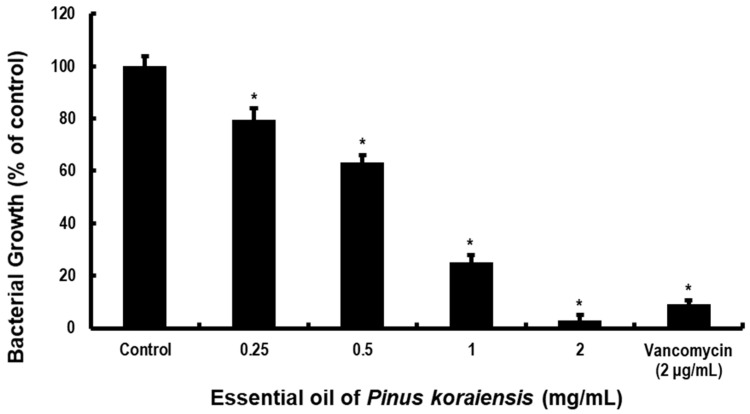
An essential oil of *Pinus koraiensis* (PKEO) inhibits the growth of methicillin-resistant *Staphylococcus aureus* (MRSA). MRSA (5 × 10^5^ CFU/well) was used to inoculate a mixture of brain–heart infusion (BHI) broth and PKEO (0.25 to 2 mg/mL) and cultured at 37 °C for 24 h. The bacterial growth was evaluated spectrophotometrically at 550 nm. Assessments were performed in triplicate and data are presented as the means ± standard deviation of the three treatments. * *p* < 0.05 values were determined against the growth of the negative control group MRSA. Positive control group MRSA were treated with 2 μg/mL vancomycin.

To determine whether PKEO can prevent the growth and acid secretion of MRSA ATCC 33591, we examined the bacterial turbidity and pH changes in the presence or absence of PKEO (at concentrations ranging from 0.25 to 2 mg/mL) using a slightly modified broth dilution methodology. We accordingly detected a bacteriostatic effect of the PKEO, characterized by a significant concentration-dependent inhibition of the growth and proliferation of MRSA (Figure 1). The inhibition rates of the PKEO against planktonic MRSA growth were 20.6% at 0.25 mg/mL, 36.6% at 0.5 mg/mL, 74.7% at 1 mg/mL, and 97% at 2 mg/mL.

Table 2 presents data showing the metabolic production of organic acids, such as lactic and acetic acid, by MRSA ATCC 33591. The pH was evaluated for cultures treated with four concentrations of PKEO (0.25, 0.5, 1, and 2 mg/mL), as well as for negative and positive control groups. After 24 h of cultivation, the pH of the negative control cultures had declined from a pre-cultivation value of 7.38 ± 0.0 to 5.94 ± 0.04, thereby indicating the heightened metabolic activity of the MRSA. Similar to the MRSA growth inhibition experiment, we detected a concentration-dependent effect of PKEO on the changes in culture pH. For MRSA treated with 1 mg/mL PKEO, we recorded at a pH of 7.09 ± 0.01 after 24 h, whereas a value 7.32 ± 0.00 was obtained following treatment with 2 mg/mL PKEO, which is comparable with the pH of 7.39 ± 0.00 obtained for the positive control group treated with vancomycin, thereby indicating highly significant antibacterial and anti-acidogenic effects. On the basis of these observations, it can thus be inferred that treatment with PKEO contributes to a marked reduction in MRSA metabolism, as indicated by the reduced release of acids.

### 2.3. Destructive Effects of PKEO on MRSA Biofilm Growth

MRSA responds to host immune reactions by forming biofilms on host surfaces, and consequently we sought to evaluate the effect of PKEO on MRSA biofilm formation using safranin staining. Similar to our analyses of planktonic MRSA growth and organic acid production, we assessed the inhibitory effects of PKEO on MRSA biofilms at a concentration range from 0.25 to 2 mg/mL.

**Figure 2:**
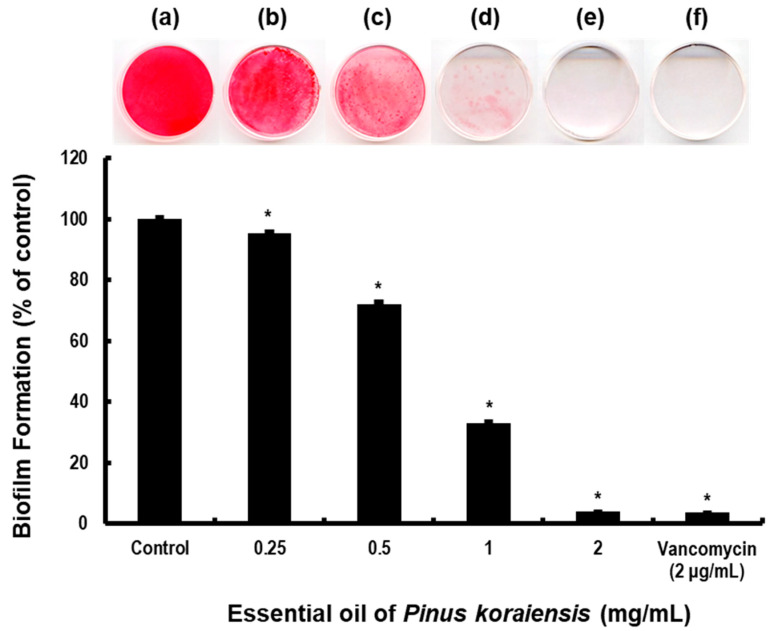
An essential oil of *Pinus koraiensis* (PKEO) inhibits the formation of MRSA biofilm. Bacteria were used to inoculate BHI broth containing 1% glucose with PKEO and incubated at 37 °C for 24 h. MRSA biofilms that had formed on the base of 35 mm Petri dishes were stained with 0.1% safranin. The safranin binding to MRSA biofilm was subsequently recovered using 30% acetic acid and the suspension thus obtained was analyzed spectrophotometrically at an absorbance at 530 nm. (**a**) Negative control; (**b**) 0.25 mg/mL PKEO; (**c**) 0.5 mg/mL PKEO; (**d**) 1 mg/mL PKEO; (**e**) 2 mg/mL PKEO; and (**f**) positive control (2 μg/mL vancomycin). Data are presented as the means ± standard deviation values obtained for three replicate treatments. Significance at the * *p* < 0.05 level was determined relative to the value obtained for the negative control group.

The effects of the PKEO on the MRSA biofilms after culturing for 24 h are shown in Figure 2. In the cases of the untreated control group, we detected a high absorbance at 530 nm, indicating the extensive safranin staining of biofilms. In contrast, we observed a progressive reduction in the staining intensity (absorbance) of biofilms following treatment with the different concentrations of PKEO. Compared with the negative control group, we detected 67% and 96.1% reductions in the staining of MRSA biofilms treated with PKEO at concentrations of 1 and 2 mg/mL, respectively, the latter value of which is comparable to the 96% reduction recorded for the vancomycin-treated positive control MRSA.

**Figure 3:**
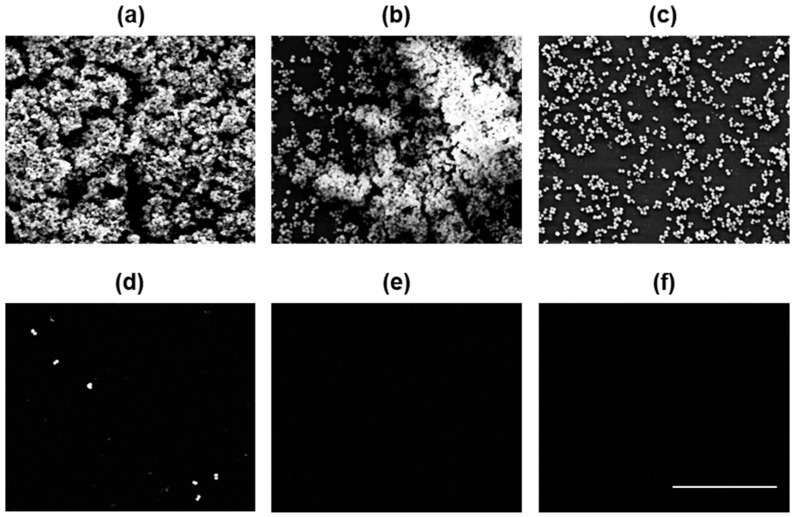
Scanning electron micrographs showing the destructive activity of an essential oil of *Pinus koraiensis* (PKEO) against MRSA biofilms. The bacteria were pre-treated with PKEO for 24 h and then immobilized in 2.5% glutaraldehyde for 2 h. After dehydrating the samples, the fixed MRSA biofilms were examined using scanning electron microscopy. (**a**) Negative control; (**b**) 0.25 mg/mL PKEO; (**c**) 0.5 mg/mL PKEO; (**d**) 1 mg/mL PKEO; (**e**) 2 mg/mL PKEO; and (**f**) positive control (2 μg/mL vancomycin). Scale bar = 10 μm.

In addition to assessing the inhibitory effects of the PKEO on MRSA biofilm formation, we also examined the state and morphology of treated biofilms using scanning electron microscopy 24 h after treatment with PKEO at concentrations ranging from 0.25 to 2 mg/mL. As depicted in the micrographs shown in Figure 3, compared with the untreated group, in which MRSA colonies formed intact three-dimensional structures, at a PKEO concentration of 0.25 mg/mL, a portion of the three-dimensional structure was disrupted, and at 0.5 mg/mL PKEO, we observed complete biofilm destruction. Moreover, in cultures treated with 1 mg/mL, we detected only individual MRSA cells, whereas no cells were observed following the treatments with 2 mg/mL PKEO and 2 μg/mL vancomycin. Morphologically, we observed a PKEO concentration-dependent reduction in MRSA biofilm thickness, revealing an inhibitory effect on MRSA biofilm proliferation similar to that observed for the positive control group treated with 2 μg/mL vancomycin.

### 2.4. PKEO Has Germicidal Effects against MRSA

To assess the bactericidal effects of PKEO treatment against antibiotic-resistant *Staphylococcus* strains, we performed an analysis of the bacterial viability based on confocal laser scanning microscopy (CLSM) observations and nucleic acid binding fluorescent staining. Given our scanning electron microscopy observations revealed a complete eradication of MRSA bacterial colonies at a PKEO concentration of 2 mg/mL, bactericidal evaluation was performed using PKEO concentrations within the range from 0.25 to 2 mg/mL.

**Figure 4:**
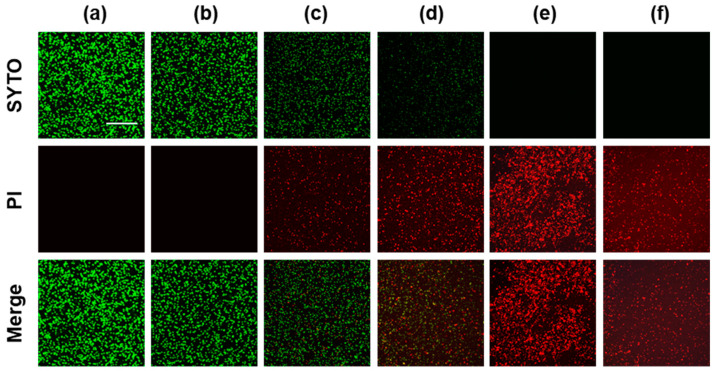
An essential oil of *Pinus koraiensis* (PKEO) reduces the viability of MRSA. Bacteria were cultured for 24 h and then treated with PKEO for 30 min. The bactericidal activity of PKEO was assessed using a LIVE/DEAD^®^ BacLight™ Bacterial Viability Kit in conjunction with confocal laser scanning microscopy (CLSM). The CLSM images revealed a concentration-dependent reduction in living MRSA stained with SYTO (green) and a concomitant increase in the proportion of dead cells stained with propidium iodide (red). (**a**) Negative control; (**b**) 0.25 mg/mL PKEO; (**c**) 0.5 mg/mL PKEO; (**d**) 1 mg/mL PKEO; (**e**) 2 mg/mL PKEO; and (**f**) positive control (2 μg/mL vancomycin). Scale bar = 50 μm.

With an increase in PKEO concentration, we detected a concentration-dependent reduction in the green fluorescence of stained MRSA and a concomitant increase in the red fluorescence of propidium iodide bound to the nucleotides in dead cells (Figure 4). In the case of the groups treated with 2 mg/mL PKEO and 2 mg/mL vancomycin (positive control), the green fluorescence signals had disappeared, and only red fluorescence was observed.

### 2.5. PKEO Represses Virulence Gene Expression in MRSA

To examine the effects of PKEO on the mRNA expression of *agrA* and *sarA*, two genes that have been established to play key roles in regulating the virulence in beta-lactam antibiotic-resistant MRSA strains, we performed real-time PCR analyses of cultures treated with PKEO at concentrations of 0.5, 1, and 2 mg/mL. Given the lack of a bactericidal effect using 0.25 mg/mL, as revealed by propidium iodide staining, we excluded this concentration from the analysis.

**Figure 5:**
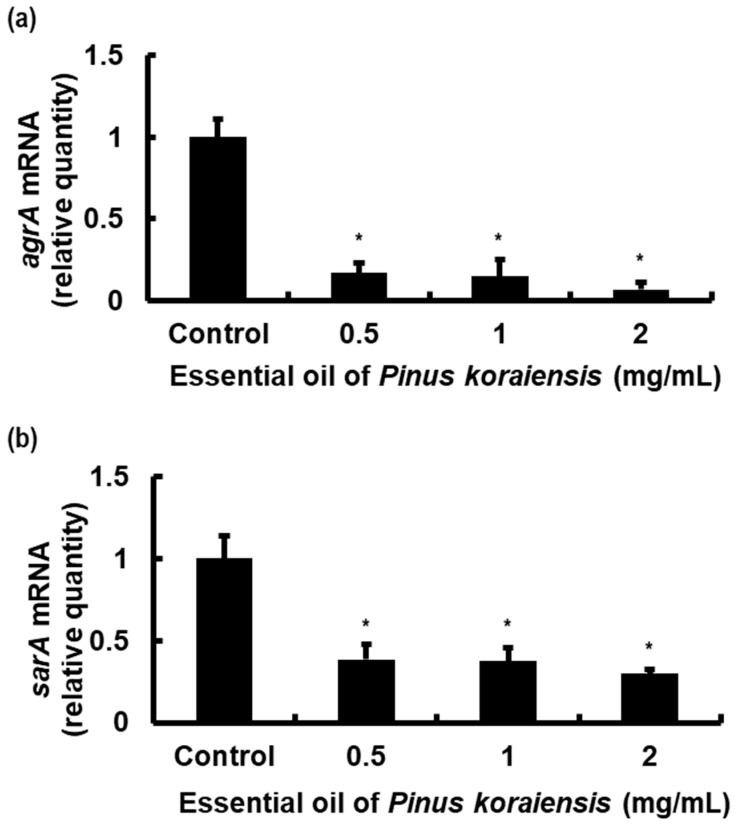
An essential oil of *Pinus koraiensis* (PKEO) inhibits mRNA expression of the MRSA virulence genes *agrA* and *sarA*. Bacterial cells were pre-treated with selected concentrations of PKEO (0.5, 1, and 2 mg/mL) for 24 h and then harvested for real-time PCR analysis. (**a**) Expression level of *agrA* mRNA, (**b**) Expression level of *sarA* mRNA. Analyses of the relative mRNA expression of virulence genes were performed in triplicate and values are presented as the means ± standard deviation of the three treatments. Significance at the * *p* < 0.05 level was determined relative to the control group.

As shown in Figure 5, compared with the negative control group, we detected a significant reduction in the mRNA expression of *agrA* in response to the treatments with the different concentrations of PKEO. A similar, although less pronounced, reduction was detected for the expression of *sarA* mRNA. We accordingly speculated that by suppressing the transcription of these virulence genes, PKEO could potentially contribute to the controlled MRSA pathogenicity.

## 3. Discussion

MRSA are commensal bacteria that are able to colonize multiple sites within the human body, including cutaneous layers, nasal cavities, and the alimentary canal [25]. Since the initial report of MRSA infections in hospitals in 1961, such infections have become globally epidemic, prevalent not only in community settings but also in livestock [26,27]. In response to the pervasiveness of MRSA infections, the efficacies of a range of antibiotic treatments have been assessed. Although vancomycin, which we used as a positive control treatment in this study, is one of the representative drugs effective in the treatment of MRSA infections, the use of this antibiotic poses risks of acute kidney injury and Redman syndrome, and moreover there is currently an increasing incidence of VRSA infections being reported [8,9,10,11]. As an effective alternative medication, we propose the administration of PKEO, which has a number of beneficial attributes. For example, *P. koraiensis* is widely distributed across much of the East Asian continent, thereby making it readily accessible. Moreover, in a number of East Asian countries, there is a long-standing tradition of the use of this plant for medicinal purposes. Consequently, it is anticipated that when used therapeutically, PKEO will have fewer side effects compared with other essential oils. Taking these factors into consideration, we accordingly sought to investigate the antibacterial activity of PKEO against MRSA ATCC 33591 cells, with a focus on its potential effects in modulating pathogenic factors.

As an initial assessment, we performed a broth dilution assay to evaluate the potential inhibitory effects of PKEO on MRSA growth. As shown in Figure 1, compared with the negative control, PKEO administered at concentrations between 0.25 and 2 mg/mL was observed to have significant inhibitory activity against planktonic MRSA growth. At concentrations of 0.25 mg/mL, 0.5 mg/mL, 1 mg/mL, and 2 mg/mL, PKEO demonstrated inhibition rates against planktonic MRSA growth at 20.6%, 36.6%, 74.7%, and 97%, respectively. The growth inhibition rate of MRSA at 2 μg/mL of vancomycin is 90.9%. In this study, a significant reduction in MRSA growth was observed at PKEO 0.25 mg/mL compared to the control group, with the inhibitory effect peaking at 2 mg/mL. The half maximal inhibitory concentration (IC50) of PKEO against planktonic MRSA was calculated as 0.68 mg/mL. This signifies that PKEO possesses sufficient potential to adequately substitute for vancomycin in inhibiting MRSA growth.

In oxygen-rich environments, *S. aureus* metabolizes glucose, yielding acetate and lactate in a 2:1 ratio, and upon glucose depletion, lactate is converted into acetate salts. Contrastingly, under anaerobic conditions, excessive amounts of lactate and minute quantities of acetate salts, ethanol, and 2,3-butanediol are generated as the end products of glucose metabolism [28]. The presence of acetic acid promotes increases in the expression of enterotoxin A, thereby leading to the development of food poisoning symptoms [29]. The extracellular matrix that constitutes MRSA biofilms primarily consists of extracellular proteins referred to as virulence factors, which include hemolysin, phenol-soluble modulins, lipase, and ribosomal proteins. Most extracellular proteins are alkaline, have a high isoelectric point (pI), and are positively charged. In conjunction with DNA, lactate and acetate salts create an electrostatically acidic environment on the bacterial surface.

In an acidic mediator environment, positively charged proteins facilitate interactions with host matrix proteins and induce bacterial aggregation [30]. In the present study, compared with the control group, comprising untreated MRSA, the treatment of cells with PKEO (0.25–2 mg/mL) was found to result in a concentration-dependent elevation in pH levels (Table 2), which would tend to indicate that PKEO can effectively inhibit the production of lactate and acetate during bacterial metabolism, hinder bacterial cell aggregation, and prevent toxin-mediated food poisoning.

On suitable surfaces, MRSA colonies form a biofilm, in which the bacteria are enveloped within an extracellular polymer matrix comprising oligosaccharides, DNA, and proteins. The development of these biofilms serves as a survival mechanism for MRSA on diverse surfaces, both biological and non-biological, leading to potential infections, particularly when present on implantable medical devices. Given the protective properties of the polymeric envelope, biofilm infections tend to have a stronger antibiotic resistance than planktonic bacteria, and are thereby a source of recalcitrant infections [31]. In the present study, we used a combination of cell culture plate spectrophotometry and scanning electron microscopy observations to evaluate the development and morphological structure of MRSA biofilms, and the efficacy of PKEO in inhibiting biofilm proliferation. When applied at concentrations of 0.25, 0.5, 1, and 2 mg/mL, we recorded percentages of biofilm inhibition of 4.6%, 27.9%, 67%, and 96.1%. Our findings that the inhibition of biofilm formation at a PKEO concentration of 1 mg/mL (67%) was slightly lower than the growth inhibition of planktonic MRSA (74.7%) can be attributed to the fact that biofilm formation can confer resistance to methicillin and enable evasion of the host’s immune response [32]. Notably, however, the inhibitory effect of 2 mg/mL PKEO on biofilm formation was found to be comparable to that of vancomycin (96.4%).

The process whereby three-dimensional MRSA biofilms develop involves four distinct phases, namely attachment, aggregation, maturation, and dispersion. Initially, planktonic bacteria adhere to a host surface via interactions mediated by surface-related proteins, following which the bacteria progressively aggregate to produce an extracellular matrix, and subsequently proliferate and accumulate to form a biofilm. As cell division proceeds, the biofilm structure becomes increasingly organized, developing a three-dimensional mushroom-like structure during the maturation stage. Finally, in response to the activity of proteases and phenol-soluble modulins (PSMs), the organized biofilm is gradually degraded, thereby enabling the bacteria to revert to a planktonic state and colonize new locations [33].

Our scanning electron microscopy observations clearly revealed a substantial reduction in the density of MRSA biofilm cells in cultures treated with increasing concentrations of PKEO, with significant concentration-dependent effects. Notably, at a concentration of 2 mg/mL, PKEO exhibited a biofilm control effect similar to that of the positive control vancomycin, suggesting its potential as a promising alternative MRSA biofilm inhibitor.

In further analyses, we evaluated the inactivation effect of PKEO on viable MRSA cells using nucleic acid-binding fluorescent dyes and CLSM. As a nucleic acid staining reagent, the SYTO™ 9 dye diffuses through the bacterial plasma membrane and emits green fluorescence upon excitation in blue [34,35]. Given the presence of a phenanthridinium group, the compound propidium iodide is unable to penetrate the cell membranes of living microorganisms, although it readily passes through the cytoplasmic and nuclear membranes of damaged bacterial cells and subsequently binds strongly to DNA and RNA as an intercalator [36]. Accordingly, our detection of a PKEO concentration-dependent reduction in green fluorescence can be taken to be indicative of a reduction in the numbers of viable cells labeled with the SYTO™ 9 dye. In contrast, the corresponding increase in red fluorescence indicates an increased number of dead cells labeled with propidium iodide. Collectively, these observations provide convincing evidence to indicate the efficacy of PKEO in reducing the viability of MRSA cells.

MRSA are equipped with an efficient adaptive system that enables these bacteria to detect and respond to signals from the external environment and host. By detecting signals, this system governs the expression of adhesion proteins located on the cell surface (e.g., protein A, cell wall secretory proteins, and surface receptors) and tissue-penetrating secreted proteins (e.g., hemolysins, lipases, and proteolytic enzymes) [37,38]. The pathogenicity regulatory system of MRSA strains comprises the pivotal genes *agrA* and *sarA*, which have specific functions [37,38,39]. *agrA* is a member of the quorum sensing system that detects and reacts with intercellular signals from bacteria in the surrounding environment [40]. In environments with a high bacterial density, *agrA* facilitates the activation of RNAII transcription from the P2 promoter, thereby enabling a self-feedback regulation of metabolic equilibrium. Most of the *agrA* molecules bind to the P3 promoter, inducing the transcription of RNAIII [37,40]. RNAIII acts as a messenger RNA that encodes the delta-hemolysin gene (delta-toxin), whilst enhancing the activation of *hla* and suppressing the biosynthesis of cell surface proteins and the repressor of toxin (Rot) protein [37,41]. *agrA* also promotes the transcription of *psm* genes and induces the secretion of the PSMα and PSMβ proteins [42]. PSM proteins impart stability by facilitating the formation of biofilms and MRSA colonization [43]. The SarA protein, encoded by the *sarA* locus, binds to the promoter regions of target genes such as aureolysin (*aur*) and the methicillin resistance gene (*mecA*), or indirectly regulates these by specifically binding to the P2 and P3 promoter sites of *agr* [43,44,45]. *sarA* contributes to the disease-causing traits of MRSA strains by regulating the expression of genes encoding hemolysin, staphylococcal enterotoxin B, and Panton–Valentine leukocidin [46,47], and has been implicated in the modulation of genes responsible for biofilm formation, including *aur*, intercellular adhesion (*ica*) A, *icaD*, surface protein A, and *fnbA* [41,46,48,49]. By inducing the mutually dependent regulatory expression of sigma factor B, which provides metabolic adaptive responses, *sarA* enhances the ability of strains to tolerate the presence of multiple antibiotics [50,51]. Our real-time PCR analyses in the present study revealed a statistically significant difference between the PKEO-treated and control groups with respect to the expression of *agrA* and *sarA* mRNA. The PKEO-mediated suppression of *agrA* and *sarA* expression in MRSA provides evidence to indicate that the efficacy of this extract is associated with an interference of the intercellular quorum sensing of these bacteria, and thereby influences the expression of genes associated with bacterial metabolism, toxicity, biofilm formation, colonization, and antibiotic resistance. Thus, our findings in this study provide direct evidence that PKEO has inhibitory effects on the pathogenicity genes *agrA* and *sarA* of MRSA.

The chemical composition of plant essential oils are variously determined by climate, geography, plant species, and physiological state [52]. On the basis of qualitative analysis using GC and GC–MS, we established that PKEO comprises 51 constituents, among which terpenoid hydrocarbons were identified as the major group of secondary metabolites. Of these, monoterpenoids (72.24%) and sesquiterpenoids (9.07%) were the most abundant terpenes present in PKEO. In terms of descending abundance, the major detected monoterpene compounds were as follows: α-pinene (21.32% of the total), α-terpineol (11.03%), *δ*-3-carene (10.32%), terpinolene (7.20%), camphene (6.22%), and limonene (5.17%). Of the sesquiterpenes detected, β-caryophyllene (4.69%) and *δ*-cadinene (4.38%) were the predominant compounds. The chemical profile of PKEO determined in this study is consistent with that reported previously in two respects. Firstly, the prevalence of terpene hydrocarbons was evident, and secondly, α-pinene, limonene, and β-caryophyllene were characterized by significant relative abundances [22,23,53]. However, in contrast to previous studies, this research identified α-terpineol, δ-3-carene, terpinolene, and camphene as the new main constituents of PKEO, excluding α-pinene, limonene, and β-caryophyllene.

The antimicrobial properties of α-pinene, limonene, and β-caryophyllene have been reported in previous studies. For example, de Araújo et al. have suggested that the monoterpenes α-pinene and limonene act as inhibitors of efflux pumps in *S. aureus* that have been established to mediate antibiotic resistance by actively transporting antibiotics out of the cell, thereby reducing intracellular concentrations [54]. Furthermore, in their assessment of the antibacterial activity of limonene, Han et al. observed no visible bacterial colony growth at a concentration of 20 mL/L [55]. Essential oils primarily consist of monoterpenes and sesquiterpenes, with the synergistic effects among these compounds resulting in the stronger antibacterial properties of mixtures compared with those of the individual major constituents [56]. Thus, it might be predicted that PKEO would have a more robust antibacterial effect than limonene administered alone. On the basis of their analyses of proteins, nucleic acids, and AKPase and PI fluorescence staining, Han et al. proposed that limonene damages the cell membrane, disrupting membrane permeability and inducing cell death. Moreover, limonene was found to reduce the membrane potential and respiratory activity of *S. aureus*, and was also shown to interfere with the function of enzymes involved in the tricarboxylic acid cycle (e.g., succinate dehydrogenase, malate dehydrogenase, and pyruvate kinase), thereby inhibiting ATPase and ATP synthesis and thus contributing to metabolic dysfunction [55]. Moreover, using flow cytometry, Yuan et al. demonstrated that treatment with β-caryophyllene for 24 h caused membrane damage in *S. aureus* [57]. Accordingly, on the basis of the findings of previous research and those reported herein, it can be speculated that α-pinene, limonene, and β-caryophyllene attenuate the expression of pathogenicity-related genes associated with cell surface proteins and bacterial metabolism in MRSA. However, further investigations will be necessary to elucidate the specific underlying mechanisms.

## 4. Materials and Methods

### 4.1. Isolation of PKEO

The samples of *P. koraiensis* were collected in August 2021 from the forest garden of Iksan Campus, Jeonbuk National University, South Korea. A sample was authenticated by Professor Byung-Kil Choo (Department of Crop Agriculture and Life Science, Jeonbuk National University, Republic of Korea). A voucher specimen (PK-2021-05) was deposited into the Laboratory of Food Chemistry, Jeonbuk National University. The needles of *P. koraiensis* were selected fresh from other plant samples and mechanically pulverized. For extraction, a 100 g sample of the pulverized material and 1 L of distilled water were placed in a 2 L round-bottomed flask and subjected to hydro-distillation for 3 h using a Clevenger-type apparatus. Proportions of the resulting PKEO were used for the GC–FID and GC–MS analyses, and the remainder was stored in amber tubes at −20 °C until used in bacterial experiments.

### 4.2. Analysis of PKEO

#### 4.2.1. Gas Chromatography–Flame Ionization Detector Analysis

Analysis of the chemical composition of PKEO was conducted using a Hewlett Packard model 6890 series gas chromatograph (Hewlett Packard, Palo Alto, CA, USA) equipped with a flame ionization detector (FID) and a DB-Wax fused silica capillary column (30 m × 0.32 mm id; 0.25 μm film thickness). The split ratio was 1:30 (*w*/*w*). Nitrogen was introduced as the carrier gas at a flow rate of 1 mL/min. The temperature program used for the GC–FID oven started at 50 °C with a subsequent gradual increase to 230 °C at a rate of 3 °C/min, at which it was held for 30 min. The injector and detector were both set at 250 °C. The relative levels of the separated components were based on the peak areas determined by integration.

#### 4.2.2. Gas Chromatography–Mass Spectrometry

Qualitative gas chromatography–mass spectrometry (GC–MS) analysis of the PKEO was performed using an Agilent Technologies 7890A gas chromatograph (Agilent Technologies, Santa Clara, CA, USA) equipped with a polar SUPELCOWAX 10 fused silica capillary column (30 m × 0.25 mm id, 0.25-μm film thickness), coupled to a 5975C mass selective detector used in the EI mode at an ionizing energy of 70 eV. The samples were analyzed at a flow rate of 1.0 mL/min using helium as the carrier gas. The thermal cycling for GC–MS was as follows. The temperature of the oven was increased from 40 °C to 230 °C at a rate of 2 °C/min, and subsequently maintained at 230 °C for 20 min. The injector and ion source were both configured at a temperature of 250 °C. The components were identified based on comparisons with the mass spectra contained in the NIST/NBS mass spectral database. Linear retention indices were manually calculated based on the analysis results computed relative to those of the *n*-paraffin (C6–C26) series [58]. The calculated retention index values were compared with those obtained using columns (DB-Wax and SUPELCOWAX 10) of similar polarity reported in the literature.

### 4.3. Bacterial Culture

The MRSA was cultured following the previously described methods [59,60]. The MRSA ATCC 33591 strain used in this study (acquired from the American Type Culture Collection; ATCC, Manassas, VA, USA) was streak-inoculated on a blood agar plate supplemented with 2.5 μg/mL oxacillin. After cultivation at 37 °C for 24 h, two or three colonies were collected using a sterile loop and transferred into brain–heart infusion broth (BHI; Difco Laboratories, Detroit, MI, USA) supplemented with 2 μg/mL oxacillin (Sigma-Aldrich, Saint Louis, MO, USA) and 2.5 μg/mL amphotericin B (Fungizone; Life Technology Co., Grand Island, NY, USA). The inoculates were incubated at 37 °C under sufficient humidity and aerobic conditions for 24 h prior to use in subsequent experiments.

### 4.4. Measurement of MRSA Growth and Acidogenicity

MRSA growth and acidogenicity were determined using a modified broth dilution method [61,62]. An MRSA culture (obtained as described in the previous section) was diluted with fresh BHI broth to a concentration of 1 × 10^8^ colony-forming units (CFU)/mL and then used to inoculate the wells of a 96-well plate (Nunc, Copenhagen, Denmark) containing PKEO (at a final bacterial density of 5 × 10^5^ CFU/mL). The PKEO was diluted two-fold to give concentrations of 2, 1, 0.5, and 0.25 mg/mL. The plates were incubated at 37 °C under ample humidity for 24 h. The MRSA cultures treated with PKEO were resuspended, and the bacterial turbidity was ascertained at 550 nm using a microplate reader (Bio-Rad Laboratories Inc., Irvine, CA, USA).

To monitor the MRSA acidogenicity, a quasi-experimental study was performed in 15 mL polypropylene tubes (PP; Corning Inc., Corning, NY, USA) using 5 mL mixtures of BHI broth and PKEO with the same MRSA density as used in the growth experiment. The acidity of suspensions was estimated using a pH meter (Corning Inc., Corning, NY, USA).

### 4.5. Determination of MRSA Biofilm Formation

The MRSA biofilms were analyzed using the biofilm assay procedure established by Seidl et al. [63]. BHI broth containing 1% glucose was prepared and filtered through a sterile 0.45 μm polyether sulfone membrane filter (Nalge Nunc International, Rochester, NY, USA) prior to use. Aliquots of BHI medium supplemented with 1% glucose and PKEO were dispensed into the wells of 12-well plates (Nunc, Copenhagen, Denmark) and in 35 mm polystyrene cell culture dishes (Corning Inc., Corning, NY, USA), followed by inoculation with MRSA (final bacterial density of 5 × 10^5^ CFU/mL) and incubation at 37 °C for 24 h. Thereafter, having thoroughly removed the supernatant, the culture products were washed three times with phosphate-buffered saline (PBS; Gibco Laboratories, Grand Island, NY, USA), and then stained with 0.1% safranin (Becton, Dickinson and Company, Sparks, MD, USA) for 30 s. The samples were then rinsed three times with sterile distilled water and allowed to dry in ambient air. After drying, the dishes were photographed and visualized. The safranin immobilized on the bacteria remaining in the 12-well plates was dissolved in 30% acetic acid (Sigma-Aldrich, Saint Louis, MO, USA), and the color intensity of the dye released from the MRSA was measured using a microplate reader at 530 nm.

To observe the morphological changes in the MRSA biofilms, MRSA biofilm cells cultured in 35 mm dishes were treated with 2.5% glutaraldehyde in 0.1 M sodium cacodylate buffer (pH 7.2; Sigma-Aldrich, Saint Louis, MO, USA) for 2 h according to the method described by Di Poto et al., with a few modifications [64]. The fixed MRSA biofilm layers were then dehydrated with an ascending ethyl alcohol series (50%, 70%, 80%, 95%, and 100%), each for 10 min. The bottoms of the dried dishes were cut and coated with platinum using an ion sputter (108 A; Cressington Scientific Instruments Inc., Watford, UK). The specimens were subsequently mounted on carbon tape and analyzed using a JSM-6360 scanning electron microscope (Jeol, Tokyo, Japan).

### 4.6. MRSA Viability Assay Using Confocal Laser Scanning Microscopy

For visualization of the bactericidal effect induced by the PKEO, the viability of MRSA was evaluated using a LIVE/DEAD^®^ BacLight™ Bacterial Viability Kit (Molecular Probes, Eugene, OR, USA) and confocal laser scanning microscope (LSM 510; ZEISS, Oberkochen, Germany). Aliquots of an MRSA suspension treated with PKEO were dispensed into 1.5 mL PP tubes (Genesee Scientific, San Diego, CA, USA) at 1 × 10^8^ CFU/mL and cultivated under the same conditions for 30 min. The culture supernatants were separated using centrifugation at 5000 rpm and 4 °C for 5 min, and having discarded the supernatants, the pellets were washed three times with PBS. The bacterial cells were stained for 15 min following the procedure outlined in the kit manual and samples of the stained cells on glass slides were examined using CLSM.

### 4.7. Real-Time PCR Assay of MRSA Virulence Factors

Mixtures of BHI medium (5 mL) and PKEO (0.5, 1, and 2 mg/mL) prepared in 15 mL PP tubes were inoculated with MRSA (final bacterial density of 1 × 10^9^ CFU/mL), and incubated aerobically at 37 °C for 24 h. For the purposes of real-time PCR, we extracted and processed RNA in accordance with the methodology previously described by Lee et al. [65]. Briefly, having discarded the culture supernatants, the cell pellets were rinsed three times with PBS. The total RNA was extracted using TRIzol^®^ reagent (Life Technologies, Carlsbad, CA, USA) for 10 min. The lysates were then purified to eliminate the fat layer and debris. The RNA thus obtained was quantified by measuring the absorbance at 260 nm using a spectrophotometer (Shimadzu Co., Kyoto, Japan). For cDNA synthesis, reverse transcription was performed using a RevertAid First Strand cDNA Synthesis Kit (Thermo Scientific, Berlin, Germany) according to the manufacturer’s protocol. The cDNA of the PKEO-treated MRSA was amplified using a StepOnePlus Real-Time PCR system (Applied Biosystems, Foster City, CA, USA) and VeriQuest™ SYBR^®^ Green qPCR Master Mix (Affymetrix, Inc., Cleveland, OH, USA). The DNA amplification reaction comprised an initial denaturation at 95 °C for 5 min, followed by 40 cycles of denaturation at 95 °C for 15 s, annealing at 60 °C for 1 min, and extension at 72 °C for 30 s. Relative quantification of the MRSA virulence factor gene expression was performed using the ΔΔCt method and calibrated based on the expression of the *16S rRNA* gene used as an internal control. The primers used to amplify the MRSA virulence factors are listed in Table 3.

### 4.8. Statistical Analysis

All data were derived from experiments performed in triplicate and are presented as the means ± standard deviation. The results of the in vitro study were evaluated using Student’s *t*-test and a one-way analysis of variance (ANOVA) using Microsoft^®^ Excel (Microsoft, Seattle, WA, USA). The threshold for statistical significance was set at *p* < 0.05.

## 5. Conclusions

In this study, in which we sought to identify a viable alternative to antibiotics for the control of MRSA infections, we demonstrated the antibacterial, antibiofilm, and metabolite secretion inhibitory effects of an essential oil extracted from the needles of *P. koraiensis* (PKEO) via hydro-distillation. Furthermore, we investigated the effects of this essential oil on the MRSA virulence factors *agrA* and *sarA*. The chemical profile analysis revealed PKEO to be an abundant source of terpene hydrocarbon compounds, and that it effectively inhibits planktonic MRSA growth and acid secretion during the metabolic phase. Moreover, this oil impedes the development of MRSA biofilms and reduces cell viability. We accordingly believe that PKEO is a promising therapeutic resource with broad-spectrum antimicrobial properties against organisms with β-lactam resistance in complex environments, which can be attributed to its ability to interfere with the mRNA expression of *agrA* and *sarA*, which are key regulators of MRSA pathogenicity.

## Figures and Tables

**Table 1:** Analysis of the volatile organic compounds derived from an essential oil of *P. koraiensis* using gas chromatography and gas chromatograph–mass spectrometry.

Compound Name	RI ^a^	Area [%]
α-thujene	1007	0.83
α-pinene	1021	21.32
Camphene	1063	6.22
n-hexanal	1085	0.07
β-pinene	1103	2.53
Sabinene	1120	0.03
δ-3-carene	1140	10.32
Myrcene	1171	4.60
α-phellandrene	1183	0.28
Limonene	1196	5.17
β-phellandrene	1211	1.73
*trans*-2-hexenal	1222	0.79
r-terpinene	1247	0.24
*trans*-β-ocimene	1257	0.06
ρ-cymene	1273	0.05
Terpinolene	1283	7.20
n-hexanol	1380	0.11
*cis*-3-hexen-1-ol	1385	0.92
β-thujone	1438	0.02
α-cubebene	1463	0.11
α-copaene	1487	0.32
Camphor	1516	0.03
iso-pinocamphone	1566	0.12
*cis*-α-Bergamotene	1569	0.24
Bornyl acetate	1576	3.85
β-caryophyllene	1591	4.69
Aromandendrene	1605	0.15
Terpinen-4-ol	1614	0.16
β-gurjunene	1617	0.13
γ-elemene	1633	0.08
α-humulene	1669	0.86
Neryl acetate	1685	0.10
α-terpinyl acetate	1692	1.28
α-terpineol	1697	11.03
Borneol	1703	0.33
α-muurolene	1725	0.47
γ-bisabolene	1762	0.99
δ-cadinene	1674	4.38
β-sesquiphellandrene	1770	0.25
α-cadinene	1792	0.14
Caryophyllene oxide	1980	0.20
Ledol	2028	0.15
α-cedrol	2109	0.08
τ-cadinol	2175	0.47
α-cadinol	2183	0.55
Spathulenol	2188	0.12
Stachene	2208	1.32
*trans,trans*-farnesol	2350	0.17
Dihydroabietanone	2480	0.12
Dodecanoic acid	2516	0.99
Phytol	2600	0.10
Total		96.47

^a^ Retention index on a polar DB-Wax column.

**Table 2:** The inhibitory effects of PKEO on MRSA acidogenicity.

Concentration [mg/mL]	pH (Before Cultivation)	pH (After Cultivation)
Control	7.38 ± 0.00	5.94 ± 0.04
0.25	7.39 ± 0.00	6.16 ± 0.02 *
0.5	7.37 ± 0.00	6.46 ± 0.03 *
1	7.37 ± 0.00	7.09 ± 0.01 *
2	7.38 ± 0.00	7.32 ± 0.00 *
2 μg/mL vancomycin	7.37 ± 0.00	7.39 ± 0.00 *

Each treatment was performed in triplicate, and the pH values are presented as the means ± standard deviation of three treatments. Significance at the * *p* < 0.05 level was determined relative to the value obtained for the negative control group. The positive control group was treated with 2 μg/mL vancomycin.

**Table 3:** Sequences of the primers used for real-time PCR amplification of methicillin-resistant *Staphylococcus aureus* virulence factor genes.

Genes	Sequence (5′-3′)	Length ^a^	Tm [°C]
*agrA*	Forward: 5′-TGATAATCCTTATGAGGTGCTT-3′ Reverse: 5′-CACTGTGACTCGTAACGAAAA-3′	22	50
*sarA*	Forward: 5′-TGTTATCAATGGTCACTTATGCTG-3′ Reverse: 5′-TCTTTGTTTTCGCTGATGTATGTC-3′	24	53
*16s rRNA*	Forward: 5′-ACTGGGATAACTTCGGGAAA-3′ Reverse: 5′-CGTTGCCTTGGTAAGCC-3′	20	52

^a^ Amplicon size in base pairs.

## Data Availability

Data are contained within the article.
